# miRTex: A Text Mining System for miRNA-Gene Relation Extraction

**DOI:** 10.1371/journal.pcbi.1004391

**Published:** 2015-09-25

**Authors:** Gang Li, Karen E. Ross, Cecilia N. Arighi, Yifan Peng, Cathy H. Wu, K. Vijay-Shanker

**Affiliations:** 1 Department of Computer and Information Sciences, University of Delaware, Newark, Delaware, United States of America; 2 Department of Biochemistry and Molecular & Cellular Biology, Georgetown University Medical Center, Washington, DC, United States of America; 3 Center for Bioinformatics and Computational Biology, University of Delaware, Newark, Delaware, United States of America; University of Chicago, UNITED STATES

## Abstract

MicroRNAs (miRNAs) regulate a wide range of cellular and developmental processes through gene expression suppression or mRNA degradation. Experimentally validated miRNA gene targets are often reported in the literature. In this paper, we describe miRTex, a text mining system that extracts miRNA-target relations, as well as miRNA-gene and gene-miRNA regulation relations. The system achieves good precision and recall when evaluated on a literature corpus of 150 abstracts with F-scores close to 0.90 on the three different types of relations. We conducted full-scale text mining using miRTex to process all the Medline abstracts and all the full-length articles in the PubMed Central Open Access Subset. The results for all the Medline abstracts are stored in a database for interactive query and file download via the website at http://proteininformationresource.org/mirtex. Using miRTex, we identified genes potentially regulated by miRNAs in Triple Negative Breast Cancer, as well as miRNA-gene relations that, in conjunction with kinase-substrate relations, regulate the response to abiotic stress in *Arabidopsis thaliana*. These two use cases demonstrate the usefulness of miRTex text mining in the analysis of miRNA-regulated biological processes.

## Author Summary

MicroRNAs (miRNAs) are an important class of RNAs that regulate a wide range of biological processes by post-transcriptional regulation of gene expression. The amount of literature describing experimentally validated miRNA targets is increasing rapidly, which poses a challenge to researchers and biocurators to stay up-to-date with the available information. Text mining methods have been used to extract miRNA-gene associated pairs and assist in curation. In this paper, we describe miRTex, a text mining system that extracts miRNA-target, miRNA-gene regulation and gene-miRNA regulation relations. We evaluate miRTex performance on two corpora, and show that the elaborate use of lexico-syntactic information and linguistic generalizations enables it to achieve the state-of-the-art performance. We have processed the all the Medline abstracts and all the full-length articles in the PubMed Central Open Access Subset with miRTex, and provide a website to access the extraction results from all the Medline abstracts. The full-scale text mining results will be a useful resource for miRNA researchers, while the miRTex tool itself can be integrated into literature-based curation pipelines. We present two use cases (for animal and plant miRNAs, respectively) that show how the full-scale text mining can be used in combination with other bioinformatics resources to gain insight into biological processes.

## Introduction

MicroRNAs (miRNAs) are a class of 21-25nt non-coding RNAs that negatively regulate gene expression via complementary pairing to mRNA. Since the first miRNA *lin-4* was discovered in *Caenorhabditis elegans* in 1993 [1], thousands of miRNAs have been identified in multiple animal and plant species as well as viruses. miRNAs have been found to regulate a wide range of cellular and development processes through gene expression suppression or mRNA degradation [2]. Identification of miRNA target genes is thus crucial for understanding the role of miRNA in various biological processes.

**Fig 1:**
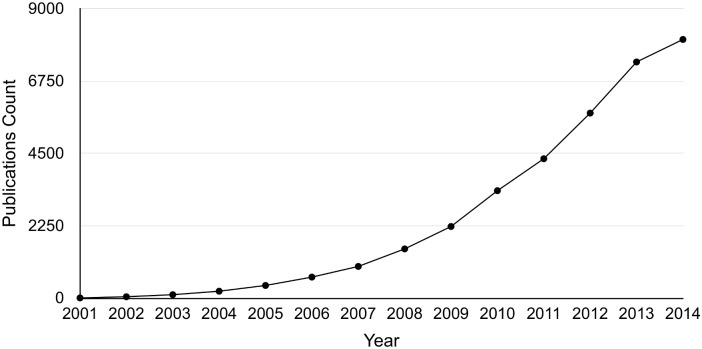
Number of ‘miRNA’ publications from 2000 to 2014 in PubMed.

Potential miRNA targets can be identified by miRNA target prediction algorithms [3–6], with limited precision [6]. Therefore, experimental validation of the predicted targets is necessary. Experimental validation methods include gene-specific techniques, e.g., reporter gene assays and assessment of miRNA and target mRNA co-expression [7], or high-throughput techniques. Validated miRNA targets are usually reported in the literature, and several databases have been built to store manually curated miRNA-target pairs, allowing researchers to access the latest results of experimentally validated miRNA targets. However, the amount of miRNA-related literature is increasing rapidly, which makes it difficult for researchers and curators to keep up to date. Fig 1 shows the trend in the numbers of papers published from the year 2001 to 2014 obtained by a PubMed search for the query ‘miRNA’ (performed in May 2015).

Significant research effort [8–10] has been made to develop automatic methods to extract relations between biological entities (e.g., gene and disease) from text. Three main kinds of methods have attracted the most attention: co-occurrence-based, rule-based and machine learning. In co-occurrence-based methods (e.g., [11–13]), the co-occurrence of entities of interest in the same sentence or paragraph is considered to indicate a relation. Co-occurrence of other types of evidences can also be used to increase extraction confidence or enrich the relation, e.g., species names, cell lines and/or experimental methods [11]. In rule-based methods, linguistics and/or biological knowledge is encoded in rules that can be implemented as an automatic program to extract relations from text. Such knowledge includes plain-text/syntactic patterns, specific words that indicate biological relations and filtering heuristics, and can be translated into deterministic steps to extract relations [14–17]. Machine learning methods view relation extraction as a classification problem and try to apply machine learning algorithms, e.g., Support Vector Machines [18] or Naive Bayes classifier [19], to extract relations. In well-studied tasks, such as protein-protein interaction extraction [20,21], or Genia Event Extraction in BioNLP [9], machine learning methods have been widely applied. Even in these settings, there have been some successful rule-based systems, such as RLIMS-P [14] and BioSEM [17]. Co-occurrence-based systems are widely used especially in emerging tasks such as mutation-disease relation extraction [13]. We have used a rule-based approach to develop miRTex, since we have developed several rule-based relation extraction systems for molecular entity relations with good results [14–16]. Furthermore, we have constructed significant infrastructure that supports generalizable and scalable development of rule-based systems [16,22].

To the best of our knowledge, only two previous papers discussed how to extract miRNA-gene associated pairs from text [11,23]. miRSel [11] detects sentences that contain both a miRNA and a gene and classifies them into one of five categories of miRNA-gene associations: physical target, repression, co-expression, induction and cleavage. This classification is based on detecting presence of words (e.g., “target” and “repress”) that indicate these types of associations anywhere in the sentence that also mentions a miRNA and a gene. An evaluation on a set of 50 abstracts containing 103 miRNA-gene associations showed a 0.78 F-score with 0.89 recall and 0.70 precision for detecting miRNA-gene associations, and a 0.73 F-score with 0.87 recall and 0.62 precision for classifying association categories.

Another text mining system [23] uses co-occurrence-based methods similar to the one used in [11] and machine learning approaches to extract miRNA-gene associated pairs. The machine learning approaches use linear classification (LIBLINEAR [24]) and linear SVM (LIBSVM [25]) with lexical features and shortest-path features from the dependency tree. Compared to the co-occurrence-based approach, the machine learning approaches performed better and achieved a 0.76 F-score with 0.67 precision and 0.87 recall on a test set of 100 abstracts with 123 miRNA-gene associated pairs. However, neither this system nor miRSel captures the direction of the extracted miRNA-gene relations, i.e., whether the miRNA regulates the gene or the gene regulates the miRNA.

In addition to these two systems that detect miRNA-gene associations from text, there are several databases containing miRNA-target information. Among them, TarBase [26], miRWalk [27] and miRTarBase [28,29] use text mining methods to assist in human curation. TarBase and miRWalk used a co-occurrence-based method that used gene names, miRNA terms and other information (e.g., interaction terms, experimental methods, pathways) to rank abstracts in a triage step. miRTarBase [28] does not provide details of the text mining method used in the triage step. Although no formal evaluation of the triage task was conducted, Vlachos et al. [26] reported that the triage step in TarBase significantly increased the efficiency of curation. The database miRecords [30] also contains experimentally validated miRNA targets, but they did not report on the use of text mining for either extraction or triage. In addition, there are other miRNA databases that are not limited to experimentally validated miRNA targets. For example, miRDB [31] contains predicted miRNA targets, miRBase [32] contains miRNA sequence information and miR2Disease [33] focuses on miRNA-disease associations.

Although text mining techniques have been used in previous works to extract miRNA-gene associations or identify potentially informative articles for curation, most of these systems used co-occurrence-based methods so, while a good performance on recall can be achieved, they generally suffer from low precision.

In this paper, we present miRTex, a text mining system that extracts regulation relations between miRNA and gene. In contrast to previous systems, miRTex uses more elaborate processing based on lexico-syntactic information and several linguistic generalizations, which we believe contributes to higher precision than the co-occurrence-based methods (see Results and Discussion Section). In addition, miRTex can capture and distinguish between a “miRNA-gene regulation” relation (i.e., the miRNA regulates the gene) and a “gene-miRNA regulation” relation (i.e., the gene regulates the miRNA), a unique aspect of miRTex to our knowledge. Furthermore, in the cases of miRNA-gene regulation relations, miRTex also detects if the miRNA-gene regulation relation is direct, i.e., the miRNA regulates the gene expression via direct binding to the gene’s mRNA. In this paper, when the miRNA-gene regulation is direct, we call this a “miRNA-target” relation. The remaining cases of miRNA-gene regulation correspond to cases where the regulation is indirect or when it is unclear from the text whether or not it is direct. The following examples elaborate the different kinds of relations we aim to extract.

**Example 1.** miRNA-gene regulation (PMID-22569260): MicroRNA-223 regulates FOXO1 expression and cell proliferation.

Here the miRNA “MicroRNA-223” regulates the gene “FOXO1”. As it is unclear whether the regulation is direct and hence we simply call this case an example of a miRNA-gene regulation relation.

**Example 2.** miRNA-gene regulation (PMID-23861775): CyclinB1, cyclinA, bcl-xl and AKt are indirectly regulated by miR-26a in a CKS2-dependent manner.

Here the miRNA “miR-26a” indirectly regulates the genes “CyclinB1” etc., and is another example of a miRNA-gene regulation relation.

**Example 3.** miRNA-target (PMID-23579289): The direct binding of miR-214 to the Osx 3' untranslated region (3' UTR) was demonstrated by a luciferase reporter assay using a construct containing the Osx 3' UTR.

Example 3 illustrates the case of a direct miRNA-gene regulation relation, i.e., a miRNA-target relation. In this example, the miRNA “miR-214” directly binds to the gene “Osx” 3' UTR. Some other examples of direct miRNA-gene regulation relations include the sentences where the gene is mentioned explicitly to be a target of the miRNA, as seen in Example 4.

**Example 4.** miRNA-target (PMID-23041385): TGF-betaR2 mRNA, a validated miR-21 target, showed the highest expression in the leukocytes from a subset of the octogenarians.

In summary, the set of miRNA-target relations is a subset of miRNA-gene regulation relations.

**Example 5.** gene-miRNA regulation (PMID-23190608): We identified Sp1, a transcription factor endowed with oncogenic activity, as a negative regulator of miR-29b expression in MM cells.

Finally, in Example 5, the gene “Sp1” is a regulator of the miRNA “miR-29b”. We call this kind of relation a gene-miRNA regulation relation.

These five examples demonstrate the kinds of relations we want to extract and illustrate some of the variety of ways in which they may be described in text. miRTex was developed to extract these relations after examination of a development literature corpus.

We created an annotated literature corpus of 350 abstracts for the development and evaluation of miRTex. 200 of these abstracts were used for development and the remaining 150 were used for evaluation. miRTex achieves F-scores close to 0.90 on this test set. We further showed that the recall of miRNA-gene regulation relation extraction can be improved by 34 percentage points (pps) by using several linguistic generalizations and relaxing a rule constraint.

We applied miRTex to the entire set of Medline abstracts as well as all the full-length articles in the PubMed Central (PMC) Open Access Subset, showing the scalability and robustness of miRTex. The full-scale processing of all the Medline abstracts also allowed us to create a database of the text mining results both for online search and file download via the website at http://proteininformationresource.org/mirtex. It is worth noting that for 75% of the full-length articles containing miRNA-gene regulation relations, miRTex extracted such relations from the full-text bodies, but not from the abstracts, indicating that full-text processing is needed for comprehensive information extraction and that miRTex is effective for full-text relation extraction.

Finally, we present two use cases that show how the text mining results from the full-scale extraction in the website can be used in combination with other tools or resources for knowledge discovery. The first use case utilizes miRTex in conjunction with gene expression data to identify candidate genes that may be regulated by miRNAs in Triple Negative Breast Cancer (TNBC, Disease Ontology [34] ID: 0060081), an aggressive form of breast cancer with limited treatment options [35]. Better understanding of the molecular mechanisms that drive TNBC, including the role of miRNAs, is crucial for the development of more effective therapy. We found that more than 35% of the miRNA-target relations extracted by miRTex, and reviewed by a curator, were not found in corresponding knowledge bases; moreover, miRTex provides valuable biological context information that is not captured by the databases. The second use case focused on the response to abiotic (non-pathogen) stress in *Arabidopsis thaliana*. Both miRNAs (e.g., [36,37]) and protein kinases (e.g., [38,39]) have been implicated in the abiotic stress response; however, the cross-talk between these regulatory mechanisms remains largely unexplored. To identify points where these regulatory pathways converge, we used miRTex results along with information from other text mining tools and databases to construct a network of miRNA-gene, kinase-substrate, and protein-protein interaction relations involving genes that have been implicated in the abiotic stress.

## Methods

miRTex extracts the miRNA-target, miRNA-gene regulation and gene-miRNA regulation relations embedded in individual sentences. Since the miRNA-target and miRNA-gene regulation mentions are far more common than the gene-miRNA regulation mentions in the literature, we describe in detail the extraction of miRNA-gene regulation relations, followed by the extraction of miRNA-target relations based on the extracted miRNA-gene regulation relations. The extraction of gene-miRNA regulation relations requires only minor modification in the system.

Typically, the regulation relation is indicated in the sentence by words such as “regulate”, “target” or “suppress”. We call these “trigger words” in this paper, because they trigger the extraction of a miRNA-gene pair by miRTex. We want to extract the “agent”, i.e., the object that performs the regulation action, and the “theme”, i.e., the object that is regulated, from the sentence. The agent and theme of regulation are invariably syntactic arguments of the trigger words in the sentences. Therefore, miRTex uses lexico-syntactic rules to extract the arguments.

**Fig 2:**
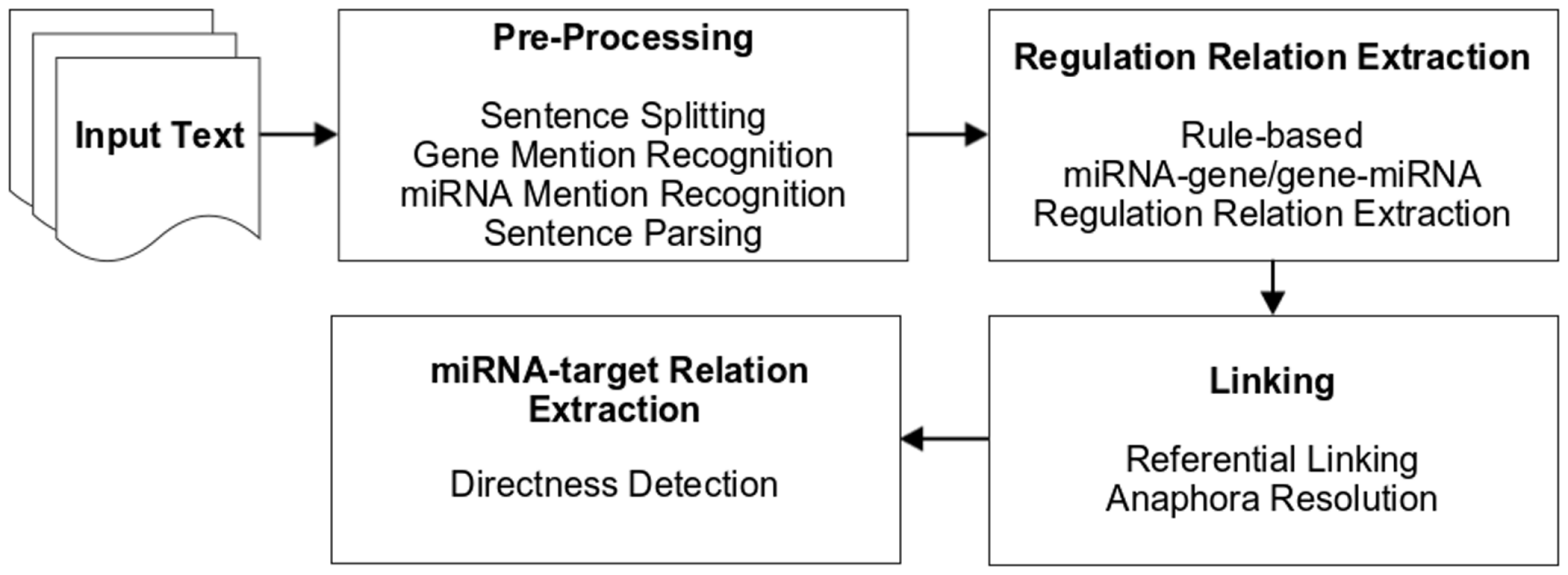
miRTex system pipeline overview.

Usually, such syntactic rule-based systems achieve high precision but have limited recall because it is difficult to manually capture all the different patterns. In miRTex, we improve the recall in two ways. First, we adapted ideas from the relation extraction systems iXtractR [16] and RLIMS-P [14], and used several linguistic generalizations to expand the coverage of the system. Second, we relaxed a constraint of the lexico-syntactic rules to extract more cases without a loss in precision. The overview of the pipeline is shown in Fig 2.

The individual components will be described in the following subsections.

### Pre-processing

In pre-processing, we first split text into individual sentences and recognize named entities, i.e., miRNA and gene. We use the Stanford sentence splitter [40], and a well-known name detector, BANNER [41], for gene mention. Unlike gene mention detection, miRNA mention detection is an easier task because miRNA nomenclature is well defined [42,43] and closely followed in the literature. The basic pattern of miRNA mentions is one prefix “miR” (or “miRNA”, “microRNA”) followed by a unique identifying number, which is assigned based on sequence similarity (e.g., “miR-1”). Additionally, the name might include one suffix such as “-a”, “-1”, “-3p” or “-5p”, and/or include a prefix that denotes the species. Most systems, including miRTex, use simple regular expressions to identify miRNA mentions.

Following name detection, parse trees of the sentences are generated to match with our lexico-syntactic rules. In this paper, we used the Charniak-Johnson parser with David McClosky’s biomedical model [44,45].

### Regulation relation extraction

#### Lexico-syntactic rules

We use lexico-syntactic rules to extract miRNA-gene regulation relation from parse tree. The rules are based on trigger words (S1 Table), a manually selected set of words that indicate regulation of a gene by a miRNA. The set of trigger words are formed by the verbs and their nominal and adjective forms in the most frequently used trigger words indicating regulation relation from the BioNLP 2013 GE [9] corpus, and the trigger words seen in our development corpus. The verbs are either words that indicate regulation, such as “regulate”, “increase”, “mediate” and “suppress”, or specific action verbs that indicate how a miRNA interacts with a gene product, such as “target”, “cleave” and “bind”.

A rule contains a syntactic pattern with typing constraints. The syntactic pattern is used for matching against the parse tree of a sentence. It expresses the syntactic constraints between the trigger word and one of its two arguments. Tree regular expressions [46] are used to match the syntactic patterns against the parse trees in our system. Since we are interested in a very specific relation, the two arguments will be agent and theme of the trigger words in all our rules. Additionally, since we are interested in the regulation of a gene by a miRNA, typing constraints require that the agent must be a miRNA and the theme must be a gene. To extract gene-miRNA regulation relations, the only necessary modification is to switch the typing constraints of the agent and theme.

Instead of a rule extracting the two arguments simultaneously, our rules only capture one argument related to the trigger at a time. Thus two rules will be needed to capture both arguments for a given sentence with the requirement that the trigger words of both rules are the same. This one-argument-per-rule design significantly simplifies the rules and reduces the number of rules that is required [14].

Although we said the two arguments are a miRNA and a gene, they can actually be noun phrases that refer to these entities or to their expression. Consider Example 6:

**Example 6.** (PMID-19487542): Taken together, these results suggested that hsa-miR-222 **regulates** the MMP1 expression.

In all of our examples, trigger words are bolded and entities are underlined. In this example, the trigger word is “regulates” and has two syntactic arguments, the subject “hsa-miR-222” and the object “the MMP1 expression”. Since the verb is in active form, these two syntactic arguments would correspond to the semantic arguments, agent and theme, respectively. A lexico-syntactic rule “agent: NP-VP” (S1 Table) contains the syntactic pattern for the verb phrase headed by an active verb and its noun phrase subject. The rule also requires that this noun phrase be of type miRNA and the head verb be “regulate” (in any tense). Matching the parse tree for the given sentence against the pattern and the constraints in this rule will allow us to extract “hsa-miR-222” as the agent of regulation. Similarly, another rule “theme: NP-VP” (S1 Table) can extract the theme “the MMP1 expression”. Because in regulation relations the argument could be the gene itself or expression of the gene, we actually extract “MMP1” as the theme. Since the trigger word for both rules applied is the same, the system extracts the regulation relationship between “hsa-miR-222” and “MMP1”.

While the above rules capture the relation using the trigger word “regulate” in the active form, the relation can be specified using other forms of the same trigger word. For example, it could also be expressed in a noun phrase “regulation of the gene by the miRNA”. The lexico-syntactic rule and tree regular expression are closely related to those for the verb “regulate” and are predictable as in our previous work iXtractR [16] and RLIMS-P [14]. An example of such a noun phrase is shown below.

**Example 7.** (PMID-23303397): **regulation** of RhoG expression by the microRNA miR-124

“RhoG” can be extracted as the theme of the trigger word “regulation” using a rule “theme: NP-of-NP-1” (S1 Table). The agent, “miR-124”, can be extracted by another rule “agent: NP-by-NP” (S1 Table).

In the above case, the nominal form of the trigger word is obtained from the verb by using the suffix “-ion”. However, sometimes more than one nominal form exists for a verb, such as the nouns “targeting” and “target” for the verb “target”. “Targeting” as a noun is similar to “regulation” in the sense that it represents the process specified by the verb. The pattern for “targeting” will bear the same relation to the verb as “regulation” to “regulate”. On the other hand, the second noun form “target” represents an entity rather than a process. Unlike “regulation” or “targeting”, in the expression “target of X”, X is the agent. Given the expression “target of X”, we take the trigger word “target” itself as the theme. This might not appear to be productive because we are not extracting the gene that is the actual theme of the targeting with this rule. However, we will show later that through a process we call referential linking, the actual target gene might be found. The idea of dealing with multiple noun forms of a trigger word is not present in iXtractR [16] but a similar method can be found in RLIMS-P [14].

We also consider trigger words that are of adjective form. Similar to the noun form case, the rule for the adjective form can be easily predicted given the rule for the original verb form.

#### Null-argument rules

We now discuss a few additional rules where one of the two arguments is not directly syntactically related to the trigger word. We take an example [14] involving phosphorylation to illustrate this ind of expression.

**Example 8.** (PMID-11780335): LMP1 activated NF-kappa B via **phosphorylation**.

Assume the task is to extract two arguments of phosphorylation: the agent “LMP1” and the theme “NF-kappa B”. As discussed in [14], the arguments of the trigger word “phosphorylation” are omitted because they are the same as those for the preceding predicate “activate”. Repeating the arguments—“LMP1 activated NF-kappa B via phosphorylation of NF-kappa B by LMP1”—is awkward and unnecessary for clarity. We believe that this is a very general kind of linguistic expression and not limited to phosphorylation. It applies to any trigger words following prepositions such as “via”, “by”, “through”, etc. We have a set of rules inspired by RLIMS-P [14] and iXtractR [16] that are called null-argument rules capable to capture such cases. Thus null-argument rules can be created for every trigger word we consider. This allows extraction from sentences like the example below.

**Example 9.** (PMID-24099915): MicroRNA-31 inhibits cisplatin-induced apoptosis in non-small cell lung cancer cells by **regulating** the drug transporter ABCB9.

A null-argument rule “agent: null-arg-VP” (S1 Table) can match this example and extract the agent “microRNA-31” for the trigger word “regulating”. The theme of the same trigger word can be extracted using the rule “theme: NP-VP” (S1 Table) and thus we obtain the regulation relation between “MicroRNA-31” and “ABCB9”.

The lexico-syntactic rules can be used to extract miRNA-gene regulation relations described by not only the trigger word “regulate” but also other trigger words, such as “suppress” and “increase”. All we need to do is replace the trigger word in the syntactic pattern. In this way, we can capture the syntactic relation between the arguments and a set of trigger words and their derived forms.

### Linking

In previous subsection we described how to extract the agent and the theme of a trigger word. Consider the fragment “a target for miR-142-3p” in Example 10 below. Two rules apply to the phrase, one of which will extract “miR-142-3p” as the agent of the target relation, and the other will extract “a target” as the theme. We call such phrases as referential phrases since they do not provide the actual argument but instead refer to the actual argument.

**Example 10.** (PMID-22870299): We show that the proinflammatory cytokine interleukin 1 alpha (IL1A) is a **target** for miR-142-3p.

In Example 10, after exploiting the “is-a” relation mentioned in the sentence, we can extract the actual target as “proinflammatory cytokine interleukin 1 alpha”/“IL1A”. We call the process of linking the referential phrase to the actual argument “referential linking”. Since the referential phrase can also be an anaphor (e.g., a pronoun), the linking can also take place via anaphora resolution.

#### Referential linking

As mentioned above, referential linking is the process of extracting the actual argument after a referential phrase has been identified by the rule-based extraction. The detection of an explicit is-a construction is one form of referential linking. As in iXtractR [16] and RLIMS-P [14], we also use appositives (Example 11) and member-collection relation (Example 12) for referential linking.

**Example 11.** (PMID-23041385): TGF-betaR2 mRNA, a validated miR-21 **target**, showed the highest expression in the leukocytes from a subset of the octogenarians.

The phrase containing the theme “a validated miR-21 target” is a description of the entity specified by “TGF-betaR2 mRNA”. To detect appositives, we use the patterns from the sentence simplifier iSimp [22].

**Example 12.** (PMID-22465011): Over-expression of miR-499 in rat BM-MSCs **increased** the expression of cardiac-specific genes, such as NKx2.5, GATA4, MEF2C, and cTnI.

The theme of the trigger word “increased” is “the expression of cardiac-specific genes”, but the actual themes are the genes listed in the end of the sentence. Each of these genes can be considered to be in a member-collection relation with the phrase “cardiac-specific genes”. By using member-collection relations miRTex will find the actual themes to be the list of genes.

#### Anaphora resolution

An anaphor, e.g., a pronoun, is an expression that refers to an entity that is previously mentioned in the text. Besides referential linking, anaphora also needs to be resolved to their antecedents. The antecedent must be chosen from the recognized mentions of miRNA or gene. While in resolution, the number and typing agreement between the anaphora and its antecedent are required, and different search scopes are defined for different anaphora. Most of them are “it” or “its” and in these cases we look for single entity in the same sentence (typically the subject) whose type (miRNA or gene) is based on the anaphora’s semantic role. The use of the possessive anaphora “their” is similar except that the referred phrase must contain more than one entity. Although the pronoun “they” and “them” appear infrequently, we also look for the antecedent (which refer to multiple entities) in the same sentence. For noun phrases that begin with “these”, “those”, we look for antecedent phrases that mention multiple entities previously in the paragraph, and for the phrases that start with “one”, “two”, “three”, etc., we look for antecedents that contain the same number of entities previously in the paragraph.

**Example 13.** (PMID-20545570): the miR-21 was upregulated in 0.5 Gy-treated TK6 cells and its **target** genes programmed cell death factor 4 (hPDCD4) phosphatase and tensin homolog (hPTEN), and sprouty homolog 2 (hSPRY2) were found to be downregulated in these cells.

The anaphora “its” is resolved to the miRNA “miR-21”. They agree on the number and type and they are in the same sentence, satisfying the requirement on search scope.

Another kind of anaphora the system resolves is relative pronouns, which appear in relative clauses. A relative clause is a kind of subordinate clause marked by a relative pronoun, which co-refers to a phrase in the main clause. As for anaphora resolution above, if the relative pronoun acts as a semantic argument of a trigger word, we link it to the nearest noun phrase of the appropriate type by token in the same constituent with the relative clause in the parse tree. Consider the following sentence,

**Example 14.** (PMID-23104180): Two miRNAs, miR-15a and miR-16, which act as putative tumor suppressor by **targeting** the oncogene BCL2, have been implicated in cell cycle, apoptosis and proliferation.

In this example, the relative pronoun “which” is first detected as the semantic argument of the trigger word “targeting” by a null-argument rule. Since it is detected as the agent, we attempt to link it to the nearest noun phrase of type miRNA, and thus we capture the regulation relation between “miR-15a”/“miR-16” and “BCL2”.

### Relaxing the same-trigger constraint

We also observed miRNA-gene regulation relations are not always described by a single trigger. For example,

**Example 15.** (PMID-17891175): we have identified additional potential mRNA **targets** of one of the miRNAs, (miR-125b) that are upregulated in prostate cancer and confirmed increased expression of one of these **targets**, EIF4EBP1.

The miRNA “miR-125b” and the gene “EIF4EBP1” are in a regulation relation, but their trigger words are not the same. Recall that our rules require the arguments to have the same trigger word; thus, this example will be neglected by the system. Moreover, even if two arguments share the same trigger word, unseen pattern or parsing errors may sometimes prevent the rule-based system from extracting both arguments for the trigger word. We relax the same-trigger constraint to solve this problem.

The intuition is that if a miRNA is the agent of regulation and a gene is the theme of regulation but described by a different trigger word in the same sentence, and they are not extracted by any of the previous rules, it is highly likely that they are in regulation relation. Thus, if two entities are not considered for extraction using the same trigger word in a sentence, we relax the same-trigger constraint so that a miRNA-gene pair can be extracted even they have different trigger words. Note that the original rules with same-trigger constraint are still used to extract miRNA-gene regulation relations with high confidence, while the relaxed rules only operate on the entities that are not in relations extracted by the same-trigger rules. For example,

**Example 16.** (PMID-16784027): the miRNA (miR) -17-92 cluster has been characterized as an oncogene, while let-7 **represses** Ras and miR-15a/-16-1 **represses** Bcl-2, thereby acting as tumor suppressors.

Three miRNA-gene regulation relations can be extracted by the rules with same-trigger constraint from this sentence, i.e., “let-7” represses “Ras”, “miR-15a” represses “Bcl-2” and “miR-16-1” represses “Bcl-2”. The rules with relaxed constraint cannot extract any relations since there is only one entity “(miR) -17-92” that does not participate in the three extracted relations. If we only apply the rules with relaxed constraint, six relations will be extracted between the three miRNAs and the two genes in a combinatorial manner; however, only three of them are correct.

In our experiments, we evaluate the same-trigger rules alone and together with rules with the relaxed constraint. We prove that the system using both sets of rules obtains a higher recall without significantly compromising precision.

### miRNA-target relation extraction

When we extract miRNA-gene regulation relations, we further detect if the relation is direct, thus considered as a miRNA-target relation. Usually we can find evidence indicating the directness of the relation in the sentence from which it is extracted. Below is a list of evidence that we consider for directness of the relation.

1. The trigger word is “target”, “bind”, “interact” or one of their nominal or adjective forms.
2. The trigger word is modified by the adjectives “direct” or “immediate” or the adverb “directly” and “immediately”.
3. The relation is extracted by a null-argument rule and the gene is down-regulated. The intuition is that the null-argument structure is often used to describe direct impact on an object by another object, and since the gene is down-regulated, it is very likely that the regulation is direct.
4. The following words co-occur in the sentence from which the miRNA-gene regulation relation is extracted: “translation”, “3’UTR” or one of its variances, e.g., “3’untranslated region”. (The 3’UTR is a region of a target mRNA to which a miRNA binds.)

If one of such evidence can be detected for an extracted miRNA-gene regulation pair, the pair is assumed to be in a direct (miRNA-target) relation.

### Evaluation and corpus preparation

We evaluated miRTex on two different corpora. First, we evaluated the extraction of all three types of relations on a test set containing 150 abstracts of an in-house developed corpus. The evaluation was at the abstract level meaning that only unique miRNA-gene pairs in an abstract are counted. Second, we evaluated miRTex on Bagewadi et al.’s corpus [23]. It contains 200 and 100 abstracts for training set and test set, respectively. We inspected the training set and found that the annotated miRNA-gene pairs for specific miRNA names (SpMiR-GP relations [23]) corresponded to both miRNA-gene regulation and gene-miRNA regulation relations in miRTex. Thus, we combined miRTex results for both miRNA-gene regulation and gene-miRNA regulation relations when evaluating using this corpus. In addition, the evaluation on this corpus was at the mention level, as in [23], because this corpus annotates duplicated miRNA-gene associated pairs in abstracts.

As there is no public corpus available for comparative evaluation of the three types of miRNA-gene relation extraction, we developed an in-house corpus consisting of 350 abstracts for both system development and evaluation. First, the query ‘microRNA[TIAB] OR miRNA[TIAB] OR miR[TIAB]’ was searched in PubMed to obtain potential abstracts containing miRNA information (performed in May 2014). This gave us about 30,000 abstracts. Second, we used our miRNA mention recognizer to extract miRNA mentions in the abstracts, and randomly selected 350 abstracts from those that have at least one miRNA mention. These abstracts were further processed by BANNER [41] to annotate gene mentions. The automatically annotated miRNA and gene mentions were manually checked and corrected by an annotator. Finally, these 350 abstracts were divided into a development set of 200 abstracts and a test set of 150 abstracts and annotated by two other annotators.

Prior to formal annotation, the two annotators were asked to annotate independently a common set of 10 abstracts and compare their annotations. The aim of the comparison was to develop common annotation guidelines (S1 Dataset). Then 20 abstracts were annotated by both annotators independently based on the guidelines and the results were used to calculate the inter-annotator agreement. The remaining 320 abstracts were split into two distinct subsets and they were annotated by the two annotators individually. The annotation was conducted in an information-centric manner, i.e., only distinct miRNA-gene or gene-miRNA regulation relations were extracted from each abstract, regardless of how many times the relation was mentioned. The annotators were asked to indicate the directness of the miRNA-gene regulation, i.e., direct or unknown, from reading of the abstract. If it was direct, the miRNA-gene regulation pair was also a miRNA-target relation, in which the miRNA targets the gene directly. The characteristics of the corpus and evaluation results will be discussed in the next section.

### Full-scale processing and interactive web query

To extract the large amount of information buried in the miRNA-related literature as well as to validate the scalability and robustness of our system, we conducted two types of full-scale processing. First, we extracted relations from all the abstracts in Medline (~13M abstracts, as of May 2015). These abstracts have been downloaded (see download instructions at http://www.nlm.nih.gov/bsd/licensee/medpmmenu.html) and indexed by Lucene (http://lucene.apache.org) on our local machine. We selected the abstracts that contain specific miRNA mentions by using regular expressions to capture different forms of their mentions and then applied miRTex on this subset of abstracts. The miRTex extractions including text evidence are stored in a database called miRTexDB. Second, we applied miRTex to all the articles in the PMC Open Access Subset downloaded from http://www.ncbi.nlm.nih.gov/pmc/tools/ftp (~1M full-length articles, as of May 2015). Based on our experience with the extraction of phosphorylation information [14], we feel that it is important to process full-length text because many of the relations are mentioned in full-length text only and not present in the abstract. Additionally, a previous study [47] noted that there are differences between the text in abstracts and full-length article bodies; thus we wanted to verify the effectiveness of our system on full-length text. As before, we applied miRTex only on the subset of articles that contain at least one or more miRNA mention. Each full-length article was divided into individual sections and subsections. Each of the subsections was treated as an independent unit of text so that the scope of anaphora resolution is limited to that subsection. The statistics of the two full-scale extractions are reported in next section, including a discussion of information found in the bodies of full-length articles as compared to their abstracts.

We have developed a website for interactive query of the miRTex extractions in miRTexDB. The web interface accepts PubMed-like queries as input, supporting user queries such as a miRNA, a gene name, or even a general concept. For example, a user interested in miRNA-gene regulation in the context of Triple Negative Breast Cancer (TNBC) can search in the interface using the query ‘ “triple negative breast cancer” OR TNBC’.

**Fig 3:**
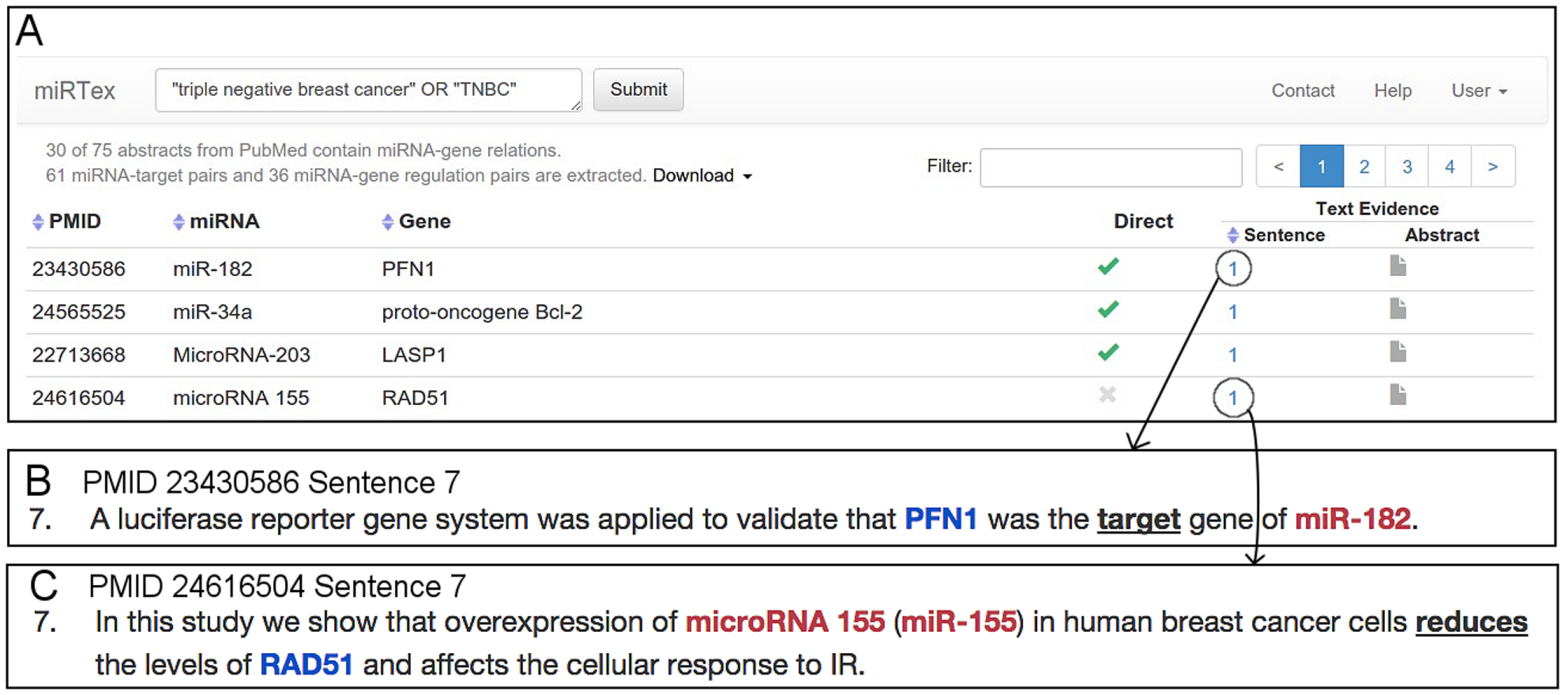
Querying miRTexDB with the keywords “triple negative breast cancer” OR “TNBC”. (A) Screenshot from the miRTex website showing a portion of the results page for the query ‘ “triple negative breast cancer” OR TNBC’. (B) Highlighted text evidence [48] for the first result indicating that PFN1 is a direct target of miRNA-182. The text evidence is obtained by clicking on the number in the Sentence column. (C) An example of text evidence sentence [49] describing a miRNA-gene regulation relation whose directness is unknown.

The query will be appended with the “AND” operator and miRNA keywords connected by the “OR” operator, i.e., ‘query AND (miRNA[TIAB] OR microRNA[TIAB] OR miR[TIAB])’, since only the abstracts containing miRNA-related information have been processed by miRTex. The system, in turn, submits the query to PubMed and retrieves the list of PMIDs that satisfy the query. Since miRTex has already processed all the Medline abstracts, the system uses the PMID list to extract the information stored in miRTexDB for these abstracts. Fig 3 shows a screenshot of the displayed result (partial) for the query ‘ “triple negative breast cancer” OR TNBC’. In total, 75 abstracts are found for the query appended with miRNA keywords, from which 30 contain extracted miRNA-gene pairs: 61 miRNA-target relations and 36 miRNA-gene regulation relations. The first three columns show the PMID, miRNA and gene name. The fourth column indicates the directness of the extracted miRNA-gene regulation relation. The final two columns allow the user to see the literature evidence for the extracted pairs, at either the sentence or abstract level. Fig 3B and 3C depict examples of sentences for two cases, a direct target relation pair and a miRNA-gene regulation relation pair, respectively. Clicking on the last column displays the entire abstract with all the miRNA-gene pairs and trigger words highlighted. Results can be sorted by PMID, miRNA, gene, or number of positive sentences by clicking on the blue arrows next to the corresponding column headings.

Note that since the results are already pre-computed and stored, no text processing is necessary for any query and hence the results are immediately available. The full results of a specific query can be downloaded as a CSV file and used for further analysis or input into another tool for further processing (e.g., enrichment analysis).

### Use cases

We present two use case studies utilizing miRTex information obtained through the website. The two use cases explore the role of miRNA-gene regulation in two scenarios: an animal disease and an environmental response in plants.

#### Study 1: Triple negative breast cancer

For our study of gene regulation by miRNAs in TNBC, we took advantage of two modes of querying miRTexDB: with keywords and with a list of genes of interest. Our first query ‘ “triple negative breast cancer” OR TNBC’ gave us the miRNA-gene regulation relations that have been specifically mentioned in the context of this disease. Our second query, to identify additional genes that may be targeted by miRNAs in TNBC, consisted of a list of genes with decreased expression in TNBC. The selected genes (252 genes total) were down-regulated at least 2-fold in TNBC relative to ER/PR/HER2-positive breast cancer with FDR < 0.05 according to Supplementary file 07 of [50]. A network featuring the TNBC-associated miRNAs from the first query and their targets from both queries was constructed using Cytoscape 3.1.1 [51]. A list of the distinct miRNA-gene regulation and miRNA-target relations from both queries is provided in S2 Table.

The results obtained using miRTex were compared with those available in TarBase 7.0, the current largest database of validated miRNA targets, and miR2Disease, a manually curated database for miRNA involvement in human disease. The databases were searched for the target genes identified by miRTex and all miRNA-gene relations involving those genes were retrieved. miRNA-gene relations identified by miRTex that were also present in TarBase were tabulated as were any diseases that were associated with miRTex miRNA-gene relations in miR2Disease. Results of these comparisons are in S2 Table. Conversely, we performed a comparison to see how many of the relations in TarBase 7.0 and miR2Disease can also be extracted by miRTex. Since TarBase 7.0 is not available for bulk download, we sampled the database by searching the 252 TNBC-downregulated genes used in our second query (above) in TarBase 7.0 to get all the miRNA-gene relations for these genes. For miR2Disease, we were able to download all the miRNA-gene relations. We retrieved the abstracts of the publications cited as evidence for each relation by TarBase 7.0 and miR2Disase, and manually identified those cases where the gene and miRNA were both mentioned in the cited abstract. Next, we calculated the recall of miRTex for the relations mentioned in those abstracts (S2 Table). In all the comparisons, cases where miRTex identified a miRNA by a more general name (e.g. miR-122) while the databases used a more specific name (e.g. miR-122-5p) were scored as a match.

#### Study 2: Abiotic stress response

To collect information on the abiotic stress response in Arabidopsis we first searched in miRTexDB with the query ‘abiotic AND Arabidopsis’ to obtain miRNA-gene regulatory relations discussed in the context of abiotic stress. Next, kinase-substrate relations relevant to abiotic stress were identified using RLIMS-P [14], which extracts mentions of kinases, substrates, and phosphorylation sites from text. The list of miRNA targets and phosphorylated proteins that we obtained from miRTex and RLIMS-P was used to search the *Arabidopsis* phosphorylation database, PhosPhAt [52] in order to find other kinases that regulate these proteins. Because the PhosPhAt search results were not restricted to kinases involved in the abiotic stress response, we next used two methods to determine their involvement in this process. First, we inspected the biological pathway annotation accompanying the kinase entries in PhosPhAt and second, we conducted PubMed searches using the query ‘kinase name AND abiotic’. Kinases with no evidence linking them to abiotic stress were still included in the network because they can provide critical missing links and may be novel players in the stress response. Kinases, phosphorylated substrates and miRNA targets identified in all of the above queries were input into STRING [53] (parameters: data sources = Experiments and Databases; confidence > 0.4) to identify protein-protein interactions linking them. Finally, we manually extracted transcription factor-target relations that were described in the same abstracts as the miRTex results. The network was constructed using Cytoscape 3.1.1 [51]. The complete list of relations in the network is provided in S3 Table.

## Results and Discussion

### Corpus characteristics

Major characteristics of our in-house annotated corpus are listed in Table 1.

**Table 1:** Development and test set.

	Development Set	Test Set
Abstracts	200	150
Unique miRNA mentions	230	181
Unique gene mentions	469	365
Abstracts with at least one related pair	107	86
Abstracts with no related pairs	93	64
miRNA-gene regulation related pairs	259	229
miRNA-target pairs	155	128
Gene-miRNA regulation related pairs	24	36
Unique miRNA-gene regulation related pairs	253	221
Unique miRNA-target pairs	152	125
Unique gene-miRNA regulation related pairs	24	36

For example, 229 miRNA-gene regulation pairs were annotated in 86 of the 150 abstracts from the test set; of these, 128 were marked as direct regulation. The corpus is available in S1 Dataset. We calculated the inter-annotator agreement in terms of F-score as was done in [54], where one of the annotators’ annotation is taken as gold standard, and the other’s annotation is contrasted with it. The inter-annotator agreement for the relations annotated on a common set of 20 abstracts is 86%.

### Evaluation results

#### Evaluation results on in-house corpus

After developing miRTex using the development set, we evaluated it by running the system on the test set. Three different settings were used to run the system: 1) the same-trigger rules only, 2) the same-trigger rules with the linking component, and 3) the full system with the same-trigger rules, the linking component and the relaxed rules. Comparison among the results produced by these three settings allows us to verify the extent of the improvement in recall by usage of the linking process as well as the relaxed rules and measure if there is any significant drop in precision. Additionally, in order to compare our system with previous co-occurrence-based methods, we also implemented a system that we believe closely follows the description of the method described in the miRSel system [11]. The results of our evaluations are listed in Table 2.

**Table 2:** Evaluation results for miRNA-gene relation extraction on the test set.

Relation Extraction	miRNA-gene regulation			Gene-miRNA regulation			miRNA-target		
System	P	R	F	P	R	F	P	R	F
co-occurrence	0.61	0.94	0.74	0.09	0.97	0.17	0.35	0.97	0.52
miRTex–LK–RR	0.96	0.57	0.72	1.00	0.53	0.69	0.95	0.46	0.62
miRTex–RR	0.96	0.79	0.87	0.94	0.83	0.88	0.96	0.80	0.88
miRTex	0.96	0.91	0.94	0.94	0.83	0.88	0.96	0.81	0.88

P, R, F stand for precision, recall and F-score, respectively; co-occurrence: our implementation based on [11]; miRTex—Linking Component (LK)—Relaxed Rules (RR): using the same-trigger rules only; miRTex—RR: using the same-trigger rules and the linking component; miRTex: miRTex full system using the same-trigger rules, the linking component and the relaxed rules.

For extraction of miRNA-gene regulation relations, both precision and recall of the full system are high and miRTex achieved the best F-score of 0.94. While the co-occurrence-based method obtained a slightly better score on recall, its precision is lower than our system’s by a large margin. For miRTex, using only the same-trigger rules yields a high precision but only half of the relations are extracted. The linking component improves the recall by 22 pps without a drop in precision. Most of the gains are due to the use of is-a relations and appositives in referential linking. Also, anaphora resolution helps extract relations that are described across sentences, which cannot be captured by any co-occurrence-based method that operates within a single sentence. The relaxing of the same-trigger constraint further increased recall by 12 pps, with the same precision.

The extraction of gene-miRNA regulation relations was also evaluated using the same three system settings. The full system performed the best with an F-score of 0.88. Similarly, the linking component improves the recall by 30 pps with a minor drop in precision. However, the usage of relaxed rules does not obtain significant improvement. This is because most false negatives are due to unseen trigger words. The co-occurrence-based method again achieved high recall but very low precision.

Next, we evaluated miRTex for extraction of miRNA-target relations. Based on our understanding, since relation terms for induction and co-expression are also used in miRSel [11], the system is intended to capture miRNA-gene regulation relations. The authors of miRSel did not explicitly differentiate between miRNA-gene regulation relations and miRNA-target relations. In case the co-occurrence-based system was designed to extract miRNA-target relations only, we also evaluated it for extracting miRNA-target pairs. We used the same three system settings as in the evaluation of miRNA-gene regulation relation extraction.

The full system achieved the best F-score, 0.88. The co-occurrence-based system again showed an excellent recall score but performed poorly in precision. Similar to the extraction of miRNA-gene regulation relations, the linking process increased the recall by 34 pps. However, the relaxing of the same-trigger constraint did not improve the recall significantly since most relations extracted by the relaxed rules are miRNA-gene regulation relations but not miRNA-target relations, and thus it contributes little to the extraction of miRNA-target relations.

#### Evaluation results on Bagewadi et al. corpus

The test set of Bagewadi et al.’s corpus contains 100 abstracts with 376 specific miRNA mentions and 123 miRNA-gene associated pairs (SpMiR-GP pairs [23]) for specific miRNA mentions. We first evaluated our miRNA mention recognizer on these 100 abstracts and obtained an F-score of 0.965 with precision 0.989 and recall 0.941 for the 376 specific miRNA mentions. [23] reported an F-score of 0.935 with precision 0.936 and recall 0.934 for the recognition of the 376 specific miRNA mentions. Evaluation of miRTex on this test set at the mention level gave performance of 0.87 F-score with 0.92 precision and 0.82 recall for the 123 miRNA-gene associated pairs. We also evaluated the co-occurrence method implemented based on [11] on this test set and it showed a performance of 0.71 F-score with 0.55 precision and 1.00 recall. Bagewadi et al. [23] reported 0.76 F-score with 0.68 precision and 0.87 recall for these 123 miRNA-gene relations.

### Full-scale processing

We performed full-scale processing of all the Medline abstracts and all the full-length articles in the PMC Open Access Subset. First, we extracted relations from all the Medline abstracts as of May 2015. The statistics of the extraction are listed in Table 3.

**Table 3:** Full-scale relation extraction results for Medline abstracts.

# of abstracts with mention(s) of specific miRNA	22,591
# of abstracts detected with miRNA-gene regulation relation	11,590
# of abstracts detected with miRNA-target relation	7,867
# of abstracts detected with gene-miRNA relation	2,346
unique miRNA-gene regulation pairs extracted	28,537
unique miRNA-target pairs extracted	15,716
unique gene-miRNA regulation pairs extracted	4,002

We also extracted relations from all the full-length articles in PMC Open Access Subset as of May 2015 (Table 4).

In Table 4, it is interesting to note that abstracts positive for miRNA-gene relations contain ~2.5 unique miRNA-gene regulation relations and ~2 unique miRNA-target relations on average. In contrast, a full-length article body positive for miRNA-gene relations contains ~7 unique miRNA-gene regulation pairs and ~5 unique miRNA-target relations on an average. Among the 8,618 full-length articles detected with miRNA-gene regulation relations, 6,442 (75%) do not have any relations extracted from their abstracts or titles. Instead, all relations were found only in the body of these 6,442 articles, which highlights the importance of mining from full-length articles. Additional analysis of the results reveals the information distribution in different types of sections (Table 5).

**Table 4:** Full-scale relation extraction results for PMC open access subset.

	Abstract	Full-text Body	Article
# of documents with mention(s) of specific miRNA	5,378	13,841	13,866
# of documents detected with miRNA-gene regulation relation	2,176	8,573	8,618
# of documents detected with miRNA-target relation	1,375	6,682	6,747
# of documents detected with gene-miRNA regulation relation	454	3,235	3,314
unique miRNA-gene regulation pairs extracted	5,505	61,510	63,190
unique miRNA-target pairs extracted	2,861	33,276	34,348
unique gene-miRNA regulation pairs extracted	756	7,122	7,421

The “Abstract” column contains statistics for the titles and abstracts of all the full-length articles. The “Full-text Body” column contains statistics for all the full-length articles excluding the titles and abstracts. The “Article” column contains statistics for all the full-length articles including both titles/abstracts and full-text bodies.

**Table 5:** Percentages by section type of all extracted miRNA-gene regulation pairs.

Section Type	% of miRNA-gene Regulation Pairs
Results	33.3
Discussion	20.7
Unknown Type	18.4
Figure Captions	10.2
Introduction/Background	8.6
Abstract	6.4
Methods/Materials	2.0
Conclusion	0.4

The distribution is similar to the distribution of mentions of phosphorylation as determined by RLIMS-P [14]. The full-scale processing results of Medline abstracts are available at the website as we discussed in previous section. We are in the process of making the results from full-length articles also available at the website.

### miRTex use case results

We conducted two use cases, one involving Triple Negative Breast Cancer and the other involving the response to abiotic stress in *Arabidopsis thaliana*, to demonstrate how the integration of miRTex results with the output of other text mining tools, knowledge in curated databases, and high-throughput datasets can provide insight into complex biological processes.

#### Identification of candidate miRNA-regulated genes in triple negative breast cancer

**Fig 4:**
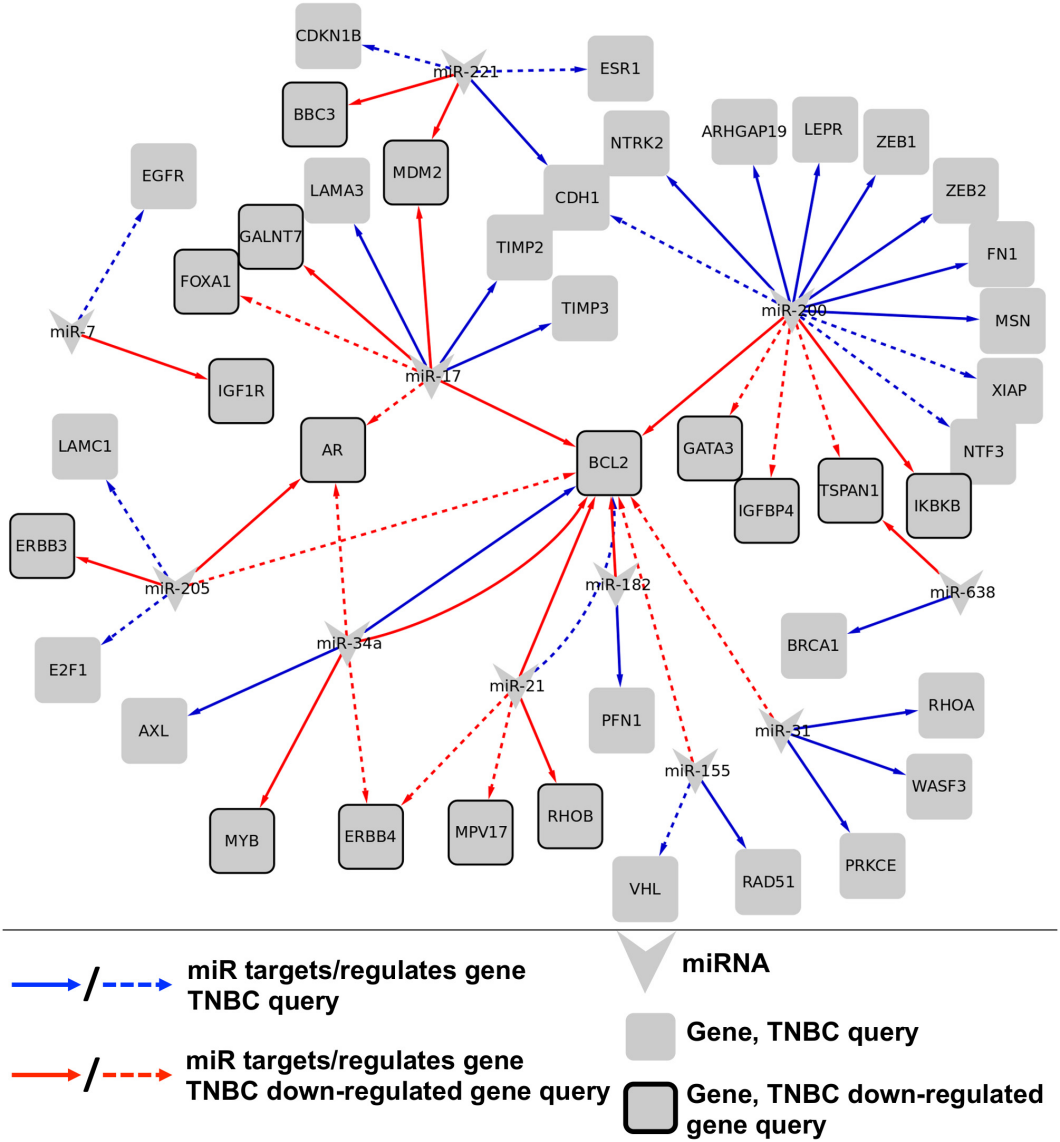
miRNA-gene relations with a potential role in TNBC. TNBC query (blue edges) refers to relations retrieved by miRTex by querying for ‘ “triple negative breast cancer” OR TNBC’; TNBC down-regulated gene query (red edges) refers to relations retrieved by miRTex by querying with a list of 252 gene down-regulated in TNBC [50]. Relations shown are those involving miRNA that appeared in the results of both queries.

In the first case study, we identified miRNA that have been mentioned in the literature in the context of TNBC and then looked for evidence of down-regulation of the targets of these miRNAs in a TNBC gene expression study. Using the query ‘ “triple negative breast cancer” OR TNBC’, we retrieved from miRTexDB 97 miRNA-gene regulation relations occurring in 30 abstracts. Results were validated by manually reviewing the accompanying highlighted text evidence. After removing false positive results (6/97 = 6%) and consolidating results that referred to the same miRNA-gene pair, there were 50 distinct miRNA-gene regulation relations that were mentioned in the context of TNBC, involving 20 distinct miRNAs and 41 distinct genes (Table 6, S2 Table). The 50 relations included 34 cases of direct miRNA-target relations (e.g., Fig 3A) and 16 cases of miRNA-gene regulation relations where the directness of the relationship was unknown (e.g., Fig 3B). A subset of these relations is shown in Fig 4 (blue edges).

Next, we checked whether these 50 relations were captured in two publicly available miRNA resources—TarBase 7.0, which houses the largest collection of experimentally verified miRNA-gene relations [26], and miR2Disease, which contains manually curated miRNA-gene relations associated with disease [33]. About two-thirds of the total miRTex relations and a similar proportion of the direct targeting events were found in TarBase (Table 6). However, if we considered the subset of relations extracted by miRTex from the more recent articles (July-December 2014), only ~30% were present in TarBase. Approximately 20% of the miRNA-gene relations extracted were associated with disease in miR2Disease, and most were associated with breast cancer, although not specifically with TNBC.

**Table 6:** Comparison between miRTex results and two miRNA-target databases.

**TNBC Articles**				
	miRTex	TarBase	miR2Disease (Disease Associated)	miR2Disease (Breast Cancer Associated)
miRNA-Gene Regulation Relations	50	34 (68%)	11 (22%)	9 (18%)
miRNA-Target Relations	34	22 (65%)	6 (18%)	5 (15%)
**TNBC Down-Regulated Genes**				
	miRTex	TarBase	miR2Disease (Disease Associated)	miR2Disease (Breast Cancer Associated)
miRNA-Gene Regulation Relations	169	81 (48%)	12 (7%)	6 (4%)
miRNA-Target Relations	101	62 (61%)	8 (8%)	4 (4%)

Number of unique miRNA-gene relations retrieved by searching in miRTexDB with the query ‘ “triple negative breast cancer” OR TNBC’ (TNBC Articles) or with a list of 252 genes reported to be down-regulated in TNBC in a published study [50] (TNBC Down-Regulated Genes). The TarBase and miR2Disease columns indicate the number (percentage) of the miRTex-identified relations that were also found in these databases.

In a second query, we used the miRTex website to identify miRNA-gene regulation relations involving the 252 most significantly down-regulated genes in TNBC reported by [50]. We found 169 distinct miRNA-gene regulation relations, involving 130 different miRNAs and 30 different genes (Table 6, S2 Table). There were 101 cases of direct targeting relationships and 68 cases of other regulatory relationships. About half of the total relations and 61% of the direct targeting relations were captured in TarBase (Table 5). Again, if we considered only relations from the more recent articles, the proportion of relations in TarBase was significantly lower (38%). Less than 10% of the relations were associated with disease in miR2Disease.

Conversely, we performed comparisons to determine the percentage of miRNA-gene relations in miR2Disease and TarBase 7.0 that can also be extracted by miRTex. For miR2Disease, we were able to download 758 miRNA-gene relations associated with 461 PMIDs from its website. In some cases, the same relation was associated with multiple PMIDs, yielding a total of 926 miRNA-gene relation citations. Because miRTex can only detect a relation between a gene and a miRNA when both are mentioned together in the text, we screened the 461 PMIDs to identify those where a gene and miRNA were both mentioned in the abstract. There were 198 such PMIDs. We manually found 329 relations were mentioned in these 198 abstracts, and 293 (89%) of the 329 relations were extracted by miRTex.

To compare with TarBase 7.0, we searched for the 252 most significantly down-regulated genes in TNBC reported by [50] in TarBase 7.0 and obtained 4,400 unique miRNA-gene relations associated with 222 PMIDs. In some cases, the same relation was associated with multiple PMIDs, yielding a total of 5,747 miRNA-gene relation citations. The great majority of TarBase 7.0 relations are derived from high-throughput experiments (~500,000 vs. 7,500 from low-throughput experiments [26]), which are likely to be reported only in supplementary data tables or in on-line repositories. Indeed, based on the information provided by the TarBase 7.0 website, of the 4,400 unique miRNA-gene relations, we found that 3,791 (86%) were detected only in high-throughput experiments; for another 478 relations (11%), the TarBase 7.0 website listed the experiment type as “other”, so we could not readily determine whether or not it was high-throughput. Thus, it is possible that as few as 3% of the relations are directly mentioned in the article text, either in the abstract or the article body. As described above for miR2Disease, we evaluated the recall of miRTex for the TarBase results, focusing on the 119 PMIDs where a gene and miRNA were both mentioned in the abstract. We manually found 144 miRNA-gene relations were mentioned in these 119 abstracts, and 123 (85%) of these 144 miRNA-gene relations were extracted by miRTex.

These results indicate that databases such as TarBase 7.0 and text-mining tools such as miRTex have complementary roles for the comprehensive understanding of miRNA-target knowledge. On the one hand, curated databases extract large numbers of relations, most of which are found in high-throughput experiments, from tables and supplementary materials. On the other hand miRTex can be used to extract relations reported in the text of the rapidly increasing body of miRNA literature. It is especially beneficial for capturing results from newly published and other articles that have not yet been curated by databases. Recall that between one-third and one-half of the relations identified by miRTex in our two use cases were not found in TarBase 7.0. Moreover, miRTex is ideal for detection of relations from low-throughput experiments in their biological or disease contexts.

We next asked which of the miRNA-gene relations we obtained in the second query were most likely to be relevant to TNBC. The fact that a gene is down-regulated in a disease context and is known to be regulated by miRNA under some conditions, does not necessarily imply that the gene is miRNA-regulated in the disease context. However, from our first miRTex query ‘ “triple negative breast cancer” OR TNBC’ we had identified 20 miRNAs whose miRNA-gene regulation relations were associated with TNBC. We reasoned that genes that: (i) were down-regulated in TNBC in the gene expression study [50] and (ii) were targets of miRNA that were associated with TNBC in our previous query would be good candidates to be miRNA-regulated in the TNBC context. Among the 169 miRNA-gene relations involving a TNBC down-regulated gene, we found 16 such genes (Fig 4, nodes with heavy borders), which participated in 28 relations (Fig 4, red edges), involving 11 miRNAs. Only one of these 16 genes, the anti-apoptosis factor *BCL2* (UniProt [55]: P10415), was uncovered in our triple negative breast cancer/TNBC query. The other 15 genes have not been previously reported to be miRNA-regulated in the context of TNBC either in Medline abstracts detected by miRTex or in bioinformatics databases such as miR2Disease. These genes are good candidates for further evaluation to determine the role of miRNA in regulating their expression in TNBC.

#### miRNA and kinase cross-talk in the *Arabidopsis* abiotic stress response

**Fig 5:**
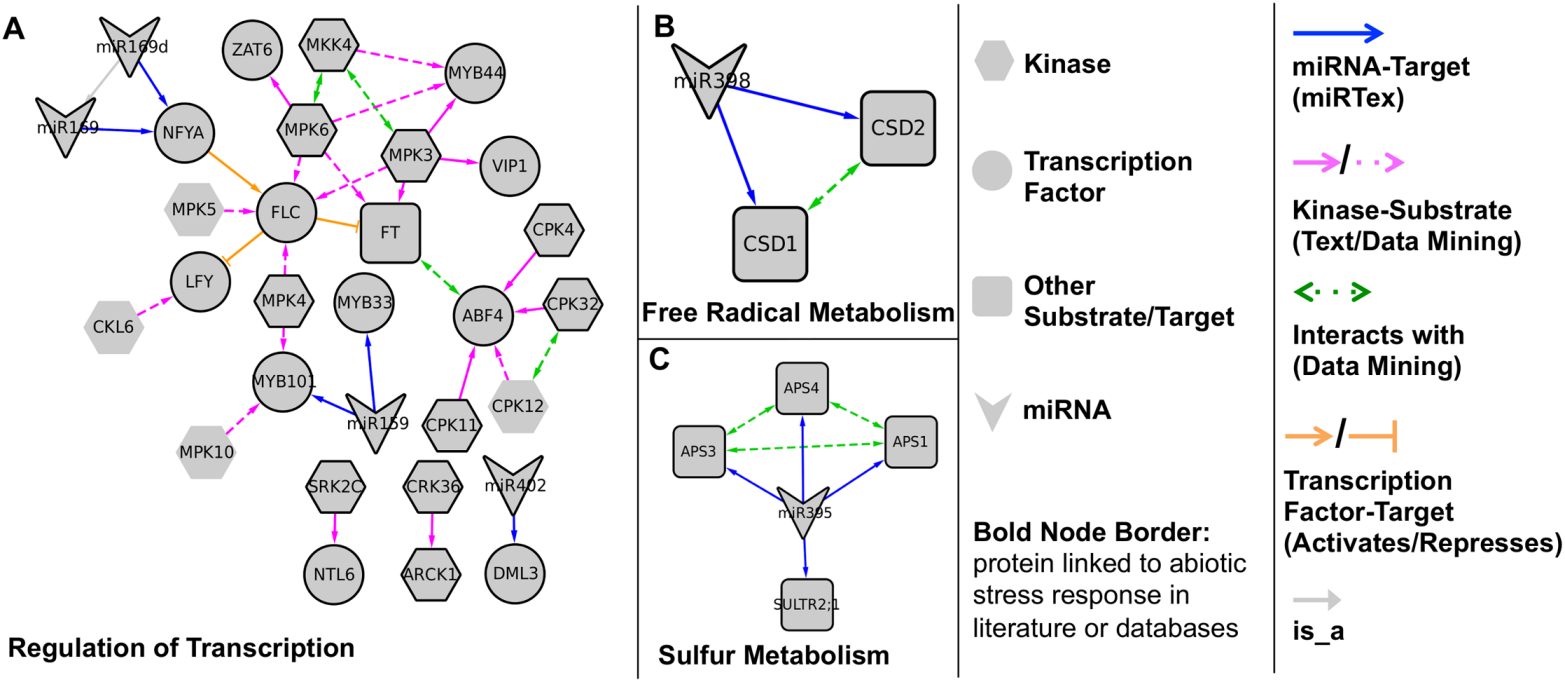
Networks showing different relations connecting genes implicated in the abiotic stress response in *Arabidopsis*. Relation types include miRNA-gene regulation, kinase-substrate, protein-protein interaction, and transcription factor-target. The predominant function of the genes in each network is indicated: regulation of transcription (A), free radical metabolism (B), and sulfur metabolism (C).

In the second study, we constructed a multi-relation network (Fig 5) to identify points where regulatory pathways converge in the *Arabidopsis* abiotic stress response. We searched in miRTexDB with the query ‘abiotic AND Arabidopsis’ to obtain abiotic-stress related miRNA-gene relations and additionally used a variety of other text mining tools and databases to capture relevant kinase-substrate and protein-protein interaction relations (see Methods Section).

Five miRNA families are represented in the network (Fig 5 and S3 Table). In three cases—miR402 targeting of the transcription factor *DML3* (UniProt: O49498) (Fig 5A), miR398 regulation of free radical metabolism (Fig 5B), and miR395 regulation of sulfur metabolism (Fig 5C), there was no evidence of co-regulation by kinases. However, the remaining two miRNAs, miR159 and miR169, are part of a complex transcriptional regulatory network that includes several kinases, including multiple members of the *mitogen activated protein kinase (MAPK)* and *calcium dependent protein kinase (CDPK)* families (Fig 5A). One of the central players in the network, *FLOWERING LOCUS C (FLC)* (UniProt: Q9S7Q7), a transcription factor that controls flowering time in response to temperature [36], is indirectly regulated by miR169 via the *NFYA* transcription factor and is a direct substrate of four MAPKs. Similarly, *MYB101* (UniProt: Q8W1W5), a transcription factor that positively regulates the response to the plant hormone abscisic acid (ABA) [37], is both a direct target of miR159 and a substrate of *MPK4* (UniProt: Q39024). These transcription factors can be experimentally tested to determine their potential role in integrating different modes of signaling during the stress response. In addition, the network includes several kinases (*MPK5* (UniProt: Q39025), *MPK10* (UniProt: Q9M1Z5), *CKL6* (UniProt: Q8LPJ1), and *CPK12* (UniProt: Q42396)) that have not been previously linked to the abiotic stress response. Their presence in the network indicates that they might be novel participants in the response to abiotic stress; their involvement can be clarified experimentally.

### Conclusion

In this paper, we described the text mining system miRTex for extraction of miRNA-target relations as well as miRNA-gene and gene-miRNA regulation relations. We combined rule-based extraction with the linking process and a relaxed same-trigger constraint to achieve high recall while preserving the high precision of the rule-based system. The system achieved the state-of-the-art performance on a test set of 150 abstracts with the evaluation results showing that the precision of our system greatly outperforms the co-occurrence-based method with a comparable recall.

The scalability and robustness of miRTex were validated by applying it to extract miRNA-gene relations from all the abstracts in Medline and all the full-length articles from the PMC Open Access Subset. The text mining results for all the Medline abstracts are stored in a database that can be searched through the website at http://proteininformationresource.org/mirtex. Two use cases studying miRNA targets in the context of Triple Negative Breast Cancer and the abiotic stress response in plants were conducted to show how to use the website and integrate the results with other bioinformatics information to address complex biological questions. The results demonstrate that miRTex extracts miRNA-gene regulation relations and miRNA-target relations not found in curated miRNA databases. Moreover, because miRTex results are extracted directly from scientific text, it is possible to make queries that identify miRNA-gene relations relevant to specific biological and disease contexts.

The corpus for system development and testing is available in S1 Dataset. We believe the release of this corpus will facilitate future comparisons of miRNA-target relation extraction systems.

With the rapid increase of the miRNA-related literature, we anticipate that the full-scale extraction results of miRTex will be a useful resource for miRNA researchers and that miRTex itself can be integrated into literature-based curation pipelines. In the future, we plan to generalize our method to extract other kinds of biomedical relations, particularly regulation-based relations.

## Supporting Information

S1 Table Lexico-syntactic rules and trigger words used in miRTex. (XLSX)

S2 Table Comparisons between miRTex results and miRNA-gene relations in TarBase 7.0/miR2Disease. The table contains miRNA-gene regulation and miRNA-target relations extracted by miRTex for the TNBC use case. The coverage of TarBase 7.0 and miR2Disease on these relations are also included in the table. Conversely, TarBase 7.0 miRNA-gene relations are sampled by searching for the 252 down-regulated genes in the TNBC use case, and all miR2Disease miRNA-gene relations were downloaded from its website. Among these TarBase 7.0/miR2Disease relations, those whose miRNA and gene can be found in the associated abstracts are included in the table. miRTex coverage on these relations are also included.
(XLSX)

S3 Table Relations presented in Fig 5. (XLSX)

S1 Dataset In-house developed corpus for development and evaluation of miRTex. The annotation guidelines are included in the dataset.
(ZIP)
