# Supplementary material for: miRTex: A Text Mining System for miRNA-Gene Relation Extraction
Source: PLoS Comput Biol. 2015 Sep 25;11(9):e1004391. doi: 10.1371/journal.pcbi.1004391 (PMC4583433; doi:10.1371/journal.pcbi.1004391)
Supplement: S1 Dataset — The annotation guidelines are included in the dataset. (ZIP) [file pcbi.1004391.s004.zip › Annotation Guidelines.docx]

**miRTex Corpus Annotation Guidelines**

MicroRNAs (miRNAs) are a class of 21-25nt non-coding RNAs that negatively regulate gene expression via complementary pairing to mRNA. Identification of miRNA target genes is thus crucial for understanding the role of miRNA in various biological processes. In this document we describe the guidelines followed for the annotation of a corpus for miRTex. The annotation is information-centric at the abstract level, i.e., only distinct miRNA-gene regulation (the miRNA regulates the gene) or gene-miRNA regulation (the gene regulates the miRNA) relations are annotated for each abstract, regardless of how many times the relation is mentioned. If a miRNA-gene regulation relation is direct then it is considered as a miRNA-target relation.

Below are examples of different relations captured:

- Example of miRNA-target relation (PMID:18185580)


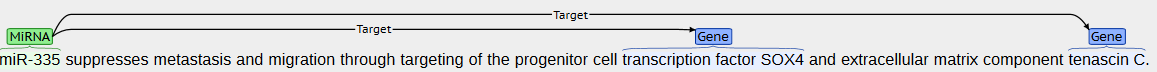


- Example of miRNA-gene regulation relation (PMID: 20498629)


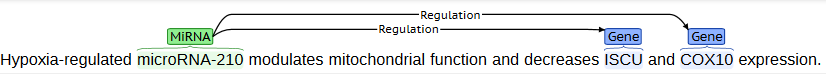


- Example of gene-miRNA regulation relation (PMID:19073597)


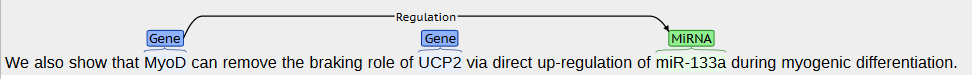


**Annotation Procedures Adopted to Annotate miRTex Corpus**

To facilitate manual annotation, the abstracts were processed using named entity recognition tools: Banner [1] was used to detect gene mentions, and an in-house tool was used to detect miRNA mentions. Abstracts are presented to the curators using the Brat rapid annotation interface (http://brat.nlplab.org). Genes are highlighted in blue and miRNAs in green. As a first pass, an annotator corrected the results of automatically recognized named entities. Gene, gene family and complex are annotated as “Gene”. miRNA and miRNA cluster are annotated as “miRNA”. If a potential entity is used as a descriptor for another entity, it is not annotated. For example, for the entity “proinflammatory cytokine interleukin 1 alpha”, “proinflammatory cytokine” is used as a descriptor for “interleukin 1 alpha”. “proinflammatory cytokine” is not annotated as a gene mention and the whole string “proinflammatory cytokine interleukin 1 alpha” is annotated as a gene mention. General descriptions of genes or miRNAs are not considered as entities, e.g., “cardiac specific genes”.

After the correction, the same annotator annotated the relations among the highlighted genes and miRNAs in all abstracts using Brat, by linking the entities involved with arrows and labeling the relations “Target” for miRNA-target relations or “Regulation”, for miRNA-gene regulation (unknown directness) and gene-miRNA regulation relations.

The following instruction text was provided to another two different annotators who were asked to review those annotations annotated by the previous annotator and delete or add missing information as needed.

**Instruction Text**

1. Review annotation of the abstracts presented in the Brat interface and identify: i) unique miRNA-gene regulation pairs (i.e., the miRNA regulates the gene), and ii) unique gene-miRNA regulation pairs (i.e., the gene regulates the miRNA). For the miRNA-gene regulation pairs, indicate whether the relation is “direct” or “unknown”. Direct miRNA-gene regulation relations are miRNA-target relations where the miRNA regulates the gene expression via direct binding to the gene’s mRNA. miRNA-gene regulation relations where the relation is indirect or no evidence in the text can be found to decide if it’s direct are annotated as “unknown” for directness. For gene-miRNA regulation relations we don’t annotate its directness.
2. Fill in a spreadsheet with the following fields.

For miRNA-gene regulation relations, the “Directness” values are “direct” or “unknown”, and the “Relation Type” values are “miRNA-gene regulation” or “miRNA-target”:

| PMID | miRNA | Gene | Directness | Relation Type |
| --- | --- | --- | --- | --- |

FFor gene-miRNA regulation relations, the “Relation Type” value is “gene-miRNA regulation”:

| PMID | Gene | miRNA | Relation Type |
| --- | --- | --- | --- |

1. Use one row per information tuple (miRNA-gene regulation or gene-miRNA regulation), except for the cases where the effect is synergistic, e.g. two genes together regulate expression of a miRNA.
2. Names of genes or miRNAs could appear in text as a combination of long names followed by the acronyms or synonyms, especially for proteins. These names should be added separated by semicolon (;) in the corresponding entity column.

| 1683187 | mir-9 | Granuphilin; Spl4 | unknown | miRNA-gene relation |
| --- | --- | --- | --- | --- |

**Annotation Guidelines**

We are interested in capturing miRNA-gene regulation and gene-miRNA regulation relations. Annotate only miRNAs or genes where some relations between them are described. Annotate miRNA mentions, gene mentions, and the type of regulation. Both miRNA and gene mentions can be single entities or families. Also, a miRNA-gene regulation relation may be direct or otherwise classified as unknown. If multiple miRNAs/genes are described for a specific relation, all should be listed in the spreadsheet. Use one line per each unique miRNA/gene pair.

Examples of miRNA-gene regulation relations are shown in the following screenshots.

In PMID 16831872,


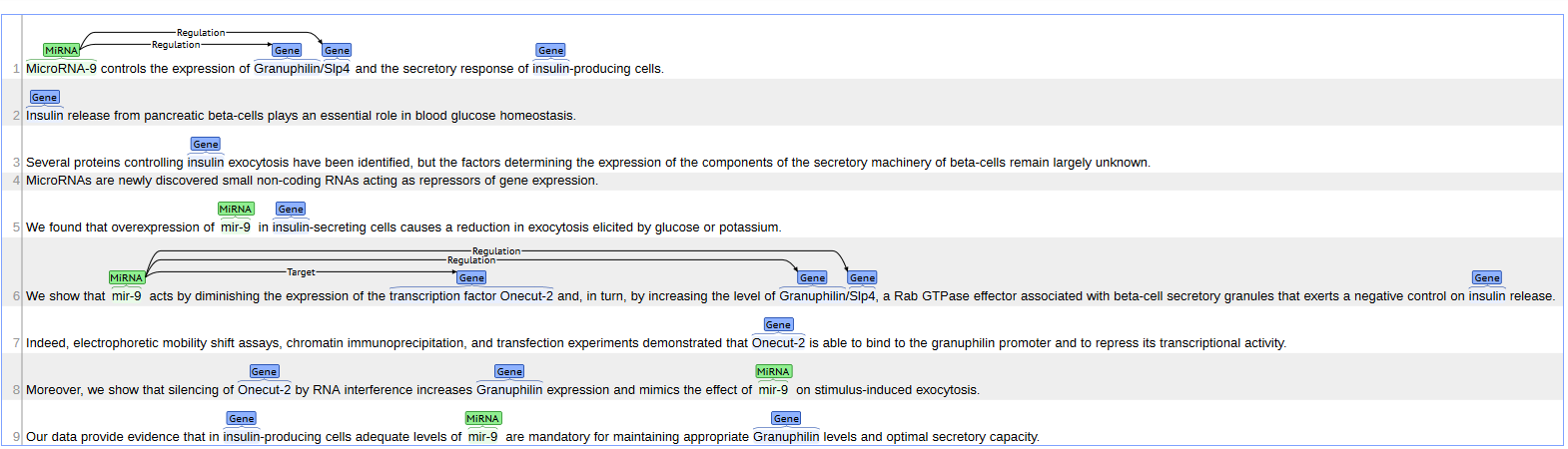


relations between mir-9 and genes can be found in sentences 1 and 6.

Sentence 1: MicroRNA-9 controls the expression of Granuphilin/Slp4 and the secretory response of insulin-producing cells.

Sentence 6: We show that mir-9 acts by diminishing the expression of the transcription factor Onecut-2 and, in turn, by increasing the level of Granuphilin/Slp4, a Rab GTPase effector associated with beta-cell secretory granules that exerts a negative control on insulin release.

In this example, there is one miRNA and two genes mentioned in the relations. The miRNA-gene regulation relation between mir-9 and Granuphilin/Slp4 should be captured in only one line of annotation and it should be labeled as “unknown” for its directness. In addition, Sentence 6 states that mir-9 is the miRNA targeting Onecut-2; this direct miRNA-gene regulation relation should be annotated on a separate line. Therefore the spreadsheet should contain the following annotation:

| PMID | miRNA | Gene | Directness | Relation Type |
| --- | --- | --- | --- | --- |
| 1683187 | mir-9 | Onecut-2 | direct | miRNA-target |
| 1683187 | mir-9 | Granuphilin; Spl4 | unknown | miRNA-gene regulation |

In PMID 20545570, there are two sentences with relations to capture.


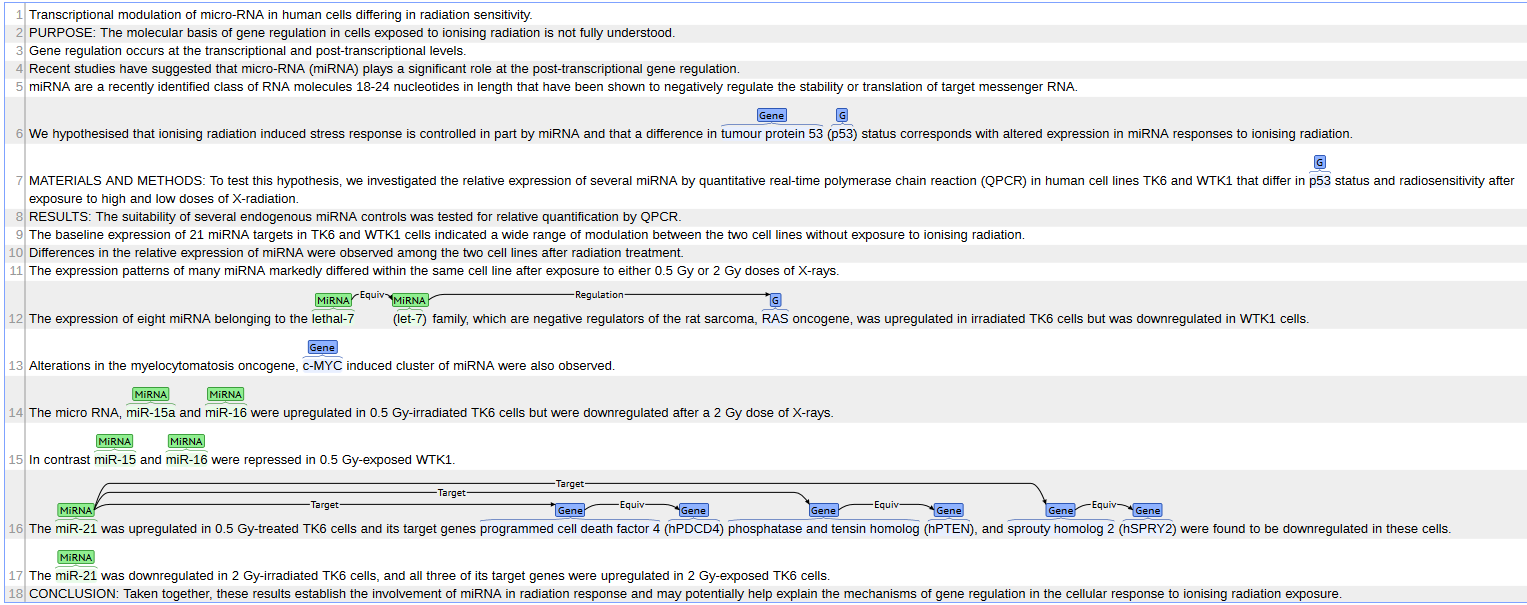


Sentence 12: The expression of eight miRNA belonging to the lethal-7 (let-7) family, which are negative regulators of the rat sarcoma, RAS oncogene, was upregulated in irradiated TK6 cells but was downregulated in WTK1 cells

Sentence 16: The miR-21 was upregulated in 0.5 Gy-treated TK6 cells and its target genes programmed cell death factor 4 (hPDCD4) phosphatase and tensin homolog (hPTEN), and sprouty homolog 2 (hSPRY2) were found to be downregulated in these cells.

The information should be summarized as follows:

| PMID | miRNA | Gene | Directness | Relation Type |
| --- | --- | --- | --- | --- |
| 20545570 | Lethal-7 (let-7) family | Ras oncogene | unknown | miRNA-gene regulation |
| 20545570 | miR-21 | Programmed cell death factor 4; hPDCD4 | direct | miRNA-target |
| 20545570 | miR-21 | Phosphatase tensin homolog; hPTEN | direct | miRNA-target |
| 20545570 | miR-21 | sprouty homolog 2; hSPRY2 | direct | miRNA-target |

In PMID 24319262,


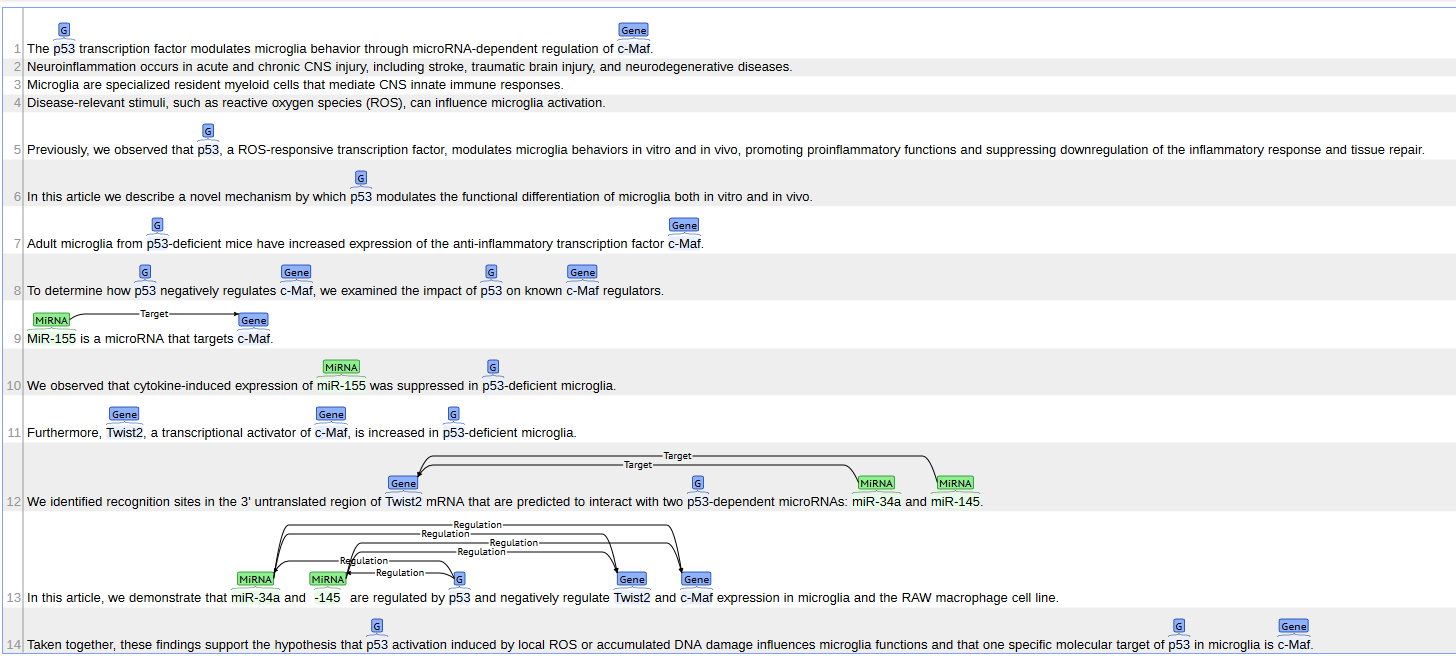


Sentence 1, 9, 12 and 13 contain relations. Sentences 1, 9 and 12 contain information about miRNA-gene regulation relations, whereas sentence 13 contains information about gene-miRNA regulation relations. The results can be summarized as follows

| PMID | miRNA | Gene | Directness | Relation Type |
| --- | --- | --- | --- | --- |
| 24319262 | MiR-155 | c-Maf | direct | miRNA-target |
| 24319262 | miR-34a | c-Maf | unknown | miRNA-gene regulation |
| 24319262 | miR-145 | c-Maf | unknown | miRNA-gene regulation |
| 24319262 | miR-34a | Twist2 | unknown | miRNA-gene regulation |
| 24319262 | miR-145 | Twist2 | unknown | miRNA-gene regulation |

| PMID | Gene | miRNA | Relation Type |
| --- | --- | --- | --- |
| 24319262 | P53 | miR-34a | gene-miRNA regulation |
| 24319262 | P53 | miR-145 | gene-miRNA regulation |

**What not to annotate**

miRNA and a gene are mentioned in a sentence but there is no relation between them.

Examples of a negative sentence

PMID: 24158791


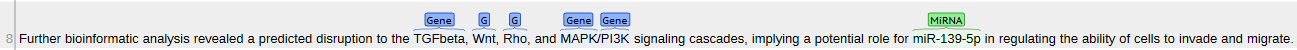


PMID: 22123611


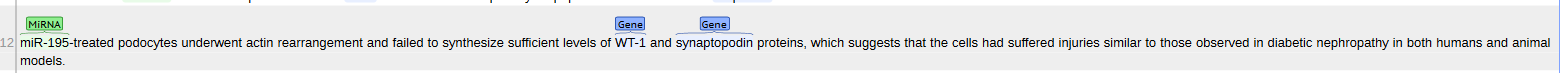


PMID: 22268758


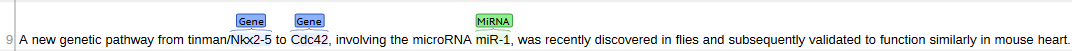


**References**

1. Leaman R, Gonzalez G (2008) BANNER: an executable survey of advances in biomedical named entity recognition. Pac Symp Biocomput: 652–663.
